# Self-rated health among older adults in India: Gender specific findings from National Sample Survey

**DOI:** 10.1371/journal.pone.0284321

**Published:** 2023-04-17

**Authors:** Saddaf Naaz Akhtar, Nandita Saikia, T. Muhammad

**Affiliations:** 1 Department of Social Work, Ben-Gurion University of the Negev, Beersheva, Israel; 2 Department of Public Health & Mortality Studies, International Institute for Population Sciences, Mumbai, India; 3 Department of Family & Generations, International Institute for Population Sciences, Mumbai, India; University of Botswana, BOTSWANA

## Abstract

**Introduction:**

The self-rated health (SRH) is a widely adopted indicator of overall health. The sponge hypothesis suggests that predictive power of SRH is stronger among women compared to men. To gain a better understanding of how gender influences SRH, this study examined whether and what determinants of gender disparity exist current self-rated health (SRH_current_) and change in SRH (SRH_change_) among older adults in Indian setting.

**Materials and methods:**

We used cross-sectional data from the 75th National Sample Survey Organizations (NSSO), collected from July 2017 to June 2018. The analytical sample constitutes 42,759 older individuals aged 60 years or older with 21,902 older men and 20,857 older women (eliminating two non-binary individuals). Outcome measures include two variables of poor/worse SRH status (SRH_current_ and SRH_change_). We have calculated absolute gaps in the prevalence of poor SRH_current_ and worse SRH_change_ by background characteristics. We carried out binary logistic regression models to examine the predictors of poor SRH_current_ and worse SRH_change_ among older adults.

**Results:**

The overall absolute gender gap in poor SRH_current_ was 3.27% and it was 0.58% in worse SRH_change_. Older women had significantly higher odds of poor SRH_current_ [AOR = 1.09; CI = 0.99, 1.19] and worse SRH_change_ [AOR = 1.09; CI = 1.02, 1.16] compared to older men. Older adults belonging to middle-aged, oldest-old, economically dependent, not working, physically immobile, suffering from chronic diseases, belonging to Muslim religion, and Eastern region have found to have higher odds of poor SRH_current_ and worse SRH_change_. Educational attainments showed lower odds of have poor SRH_current_ and worse SRH_change_ compared to those with no education. Respondents belonging to richest income quintile and those who were not covered by any health insurance, belonging to Schedule caste, OBC, Western and Southern regions are found to have lower odds of poor SRH_current_ and worse SRH_change_. Compared to those in the urban residence, respondents from rural residence [AOR = 1.09; CI = 1.02, 1.16] had higher odds of worse SRH_change_.

**Conclusions:**

Supporting the sponge hypothesis, a clear gender gap was observed in poor current SRH and worse change in SRH among older adults in India with a female disadvantage. We further found lower socioeconomic and health conditions and lack of resources as determinants of poor current SRH and its worse change, which is crucial to address the challenge of the older people’s health and their perception of well-being.

## Introduction

Aging is an unavoidable process in physiological terms. According to the World Health Organization (2020), the populations around the world are aging faster than in the past, and its demographic transition would have a significant impact on almost all aspects of society [1]. Every country throughout the world is experiencing growth in both the proportion and size of older adults in the population [2]. The primary care of older adults is mainly influenced by health services, health conditions, and socio-economic factors [3]. On the other hand, gender accentuates a pivotal role in care among the aging population with significant gaps and variations in the health conditions and the care received. Hence, the health-related gender gap in the aging process brings important health challenges and opportunities that need to be addressed. Indeed, aging healthy and successfully is a long-term goal for individuals, policymakers, and health professionals.

Self-rated health (SRH) is one of the most frequently used indicators in social, clinical, epidemiological research and also a reliable health indicator among older adults in India [4]. It is a comprehensive measure of an individual’s health status that can even reflect their condition without any clinical diagnosis [5]. Despite its non-explicit nature, it seems to be a robust predictor of future functional and physical health status, morbidity, and mortality that may differ by gender, age, place, health status, social class, culture, and countries [6, 7]. Various disease risks screening [8] and clinical trials [9] have been performed using SRH as a tool in developed countries. SRH is an individual’s subjective concept which lies between the social and biological world with psychological experiences. Generally, the empirical research on SRH arrived from the epidemiological tradition that particularly emphasized statistical associations of correlates instead of the process from which these correlations become known [7]. However, factors associated with gender gaps in current and changes in SRH status are still unclear.

Many studies emphasized that the social determinants of health outcomes, which empirically demonstrate that women, lower socioeconomic classes and low educational level have poorer health outcomes [10–18]. Apart from this, SRH also reflects psychosocial, lifestyle conditions, functional status, chronic diseases among older adults [19–22]. Another study suggested that older adults having limitations in activities of daily living, worse chronic and mental health conditions, poorer self-reported memory have lower SRH in the United States and China [23]. Studies in India revealed that older adults’ physical and functional activities had been the strong predictors in self-assessments of health [18, 19, 22]. Further, SRH is a multidimensional construct that also predicts the other health indicators such as primary health care that includes the amount of doctor visits, hospitalizations and medical tests [14, 24].

India is consistently ranked among the world’s five worst countries for female health and survival [25]. While the general public health and well-being among Indian population have been challenging, the health disparities between older men and women have not reduced significantly [26]. However, few studies have been conducted in India on SRH from a gender perspective [13, 17]. These studies have concluded that Indian women live longer but have poor SRH than men and showed a significant gender difference. While a previous study [17] also revealed that the poor SRH was observed to be greater among Muslims, Scheduled Castes, and women residing in rural areas. Earlier studies showed that gender impacts unhealthy and healthy lifestyles and gender gaps exist during health-related decision-making [27–31]. Still, SRH by gender is difficult to comprehend because of the paucity of empirical research from both the theoretical and conceptual aspects. According to the sponge hypothesis, SRH among women may absorb more information about their health problems than SRH among men and thus, SRH may reflect the health status of men and women differentially [32]. While primary determinants of SRH among men are their poor functioning and negative health behaviors, poor SRH among women is determined by their socioeconomic adversities [33, 34].

To our best knowledge, limited research has been conducted on current and changes in SRH by gender among older Indian adults. Moreover, gender can have different roles on SRH in different sociocultural settings including India and may inform policies that are region-specific [35–37]. Therefore, in the present study, our main interest is to elucidate and capture whether and how gender disparity exists in SRH_current_ and SRH_change_ in Indian settings among older adults. We also empirically assess the differences in SRH among older men and women in India based on the sponge hypothesis.

## Materials and methods

### Data source

The present study has used the data from the 25^th^ schedule of the 75^th^ round of the National Sample Survey Organizations (NSSO), collected from July 2017 to June 2018. The NSSO has been a public organization since 1950 under the Ministry of Statistics and Programme Implementation (MOSPI) of the Government of India. It is a nationally and state/Union Territory (UT) representative household, cross-sectional, population-based survey.

### Analytical sample

The analytical sample constitutes 42759 cases of older adults excluding two transgender cases. Thus, 21902 older men and 20857 older women have been considered.

### Outcome variables

The study has used two different measurements of self-rated health (SRH) among older adults. Thus, two outcome variables have been used.

○ The first outcome variable is current self-rated health. During the survey, the respondent has been asked to rate the individual’s perception about the current status of health in the last one year using the scales. The scales were categorized into three. i) Excellent, ii) fair, and iii) poor. We have categorized the response as a dummy (outcome) variable as ‘0’ indicating *‘Excellent’ and ‘1’* indicating *‘Fair’* or *‘poor’*.
○ The second outcome variable is change in self-rated health. During the survey, the respondent has been also asked to rate the individual’s perception about the change in health status in the last one year using the scales. The scales were categorized into five, i) Much better, ii) somewhat better, iii) nearly same (no change in the health status), iv) somewhat worse, and v) worse. Here, we have categorized it into a dichotomous outcome variable as a dummy, where *‘0’* indicating *‘Much better’* or *‘somewhat better’* and *‘1’* indicating *‘nearly same’* or *‘somewhat worse’* or *‘worse’*.

### Independent variables

The independent variables used in the present study mainly emphasized on socio-demographic & economic background characteristics and health information of older adults. These background characteristics comprise of age groups (in years) has three categories, such as- young-old (60–69), middle-old (70–79) and oldest-old (80+), marital status, economic dependency, educational attainment, working status, living arrangement, physical mobility status, communicable diseases, chronic diseases, any other ailments, hospitalization, insurance coverage, household income, religion, caste, household size, primary source of cooking, owned house, place of residence, regions respectively.

### Statistical analysis

We performed the univariate and bivariate analysis with suitable background characteristics. We have calculated absolute gaps in the prevalence of current own-perception and change in health status by background characteristics. The absolute gender gaps are in two folds defined as:

$${Absolute\;gender\;gaps}_{current}={\;SRH}_{current}^{older\;women}-{\;SRH}_{current}^{older\;men}$$

$${Absolute\;gender\;gaps}_{change}={\;SRH}_{change}^{older\;women}-{\;SRH}_{change}^{older\;men}$$

The study has then carried out binary logistic regression model to examine current self-rated health and change in self-rated health associations with socio-economic and demographic factors separately.

1. **Model 1** Current self-rated health status (SRH_current_,): ***‘Poor’/ ‘fair’*** *versus* ***‘Excellent’***.
2. **Model 2** Change in the self-rated health status (SRH_change_): ***‘Worse/somewhat worse’/‘nearly same’*** *versus* ***‘Much better’****/* ***‘somewhat better’***

## Results

### Sample profile

**Table 1** shows the sample profile by gender with suitable socio-economic, demographic, and health characteristics among older adults in India from the period (2017–18). There are 65.56% young-old women & 64% young-old men, with oldest-old woman (9%) somewhat higher than oldest-old men (8%), while middle-old women (25%) are lower than middle-old men (27%). Only 52% older women are currently married which is much lower than older men (84%). More than 91% older women are dependent, which is far higher than of older males (51%). Immobile older women constitute around 11% that is higher than older men (8%). About 63% of older women & 35% of older men have no education. Older women have marginally lower insurance coverage than men. Chronic disease is marginally higher among older women (24%) than older men (23%) while hospitalization cases are greater among older men (27%) than older women (24%). Majority of the older men live with spouse (83%) while only 52% of older women live with their spouse. The majority of both older women & men belonged to the rural residence, Southern region, Hindu religion, most affluent group respectively.

**Table 1:** Sample distribution of self-rated health among older adults in India by gender with suitable background characteristics, 2017–18. (n = 42,759).

Background characteristics	Men		Women	
	%	N	%	N
**Age-group (in years)**				
Young-old (60–69)	64.35	14,094	65.56	13,674
Middle-old (70–79)	27.29	5,977	25.20	5,256
Oldest-old (80+)	8.36	1,831	9.24	1,927
**Marital Status**				
Currently married	84.51	18,510	51.84	10,812
Never married	0.74	161	0.43	89
Separated or Divorced	14.75	3,231	47.73	9,956
**Economic dependency**				
Independent	48.37	10,595	8.67	1,808
Dependent	51.63	11,307	91.33	19,049
**Educational attainment**				
No education	35.37	7,746	62.92	13,123
Primary	33.05	7,238	24.67	5,145
Secondary	20.22	4,429	8.15	1,699
Higher	11.36	2,489	4.27	890
**Working status**				
Yes	51.58	11,298	67.80	14,142
No	48.42	10,604	32.20	6,715
**Living arrangement**				
With Spouse	83.16	18,214	52.32	10,913
Without Spouse	16.84	3,688	47.68	9,944
**Physical mobility status**				
Mobile	91.48	20,036	88.75	18,510
Immobile	8.52	1,866	11.25	2,347
**Communicable disease**				
No	97.71	21,401	97.75	20,388
Yes	2.29	501	2.25	469
**Chronic diseases**				
No	76.78	16,817	75.97	15,846
Yes	23.22	5,085	24.03	5,011
**Any other ailments**				
No	95.60	20,939	95.38	19,894
Yes	4.40	963	4.62	963
**Hospitalization**				
No	72.08	15,787	76.24	15,902
Yes	27.92	6,115	23.76	4,955
**Insurance coverage**				
Covered	21.08	4,616	20.42	4,258
Uncovered	78.92	17,286	79.58	16,599
**Household Income**				
Poorest	16.74	3,666	16.90	3,525
Poorer	16.54	3,622	16.90	3,525
Middle	18.96	4,153	19.06	3,975
Richer	22.65	4,960	22.40	4,673
Richest	25.12	5,501	24.74	5,159
**Religion**				
Hindus	77.52	16,979	77.96	16,261
Muslims	11.64	2,550	11.43	2,384
Christians	6.04	1,322	6.00	1,251
Others	4.80	1,051	4.61	961
**Caste groups**				
General	38.05	8,333	37.7	7,863
SC	9.21	2,018	9.09	1,895
ST	14.31	3,135	14.36	2,996
OBC	38.43	8,416	38.85	8,103
**Household Size**				
< = 5	48.20	10,556	51.03	10,644
>5	51.80	11,346	48.97	10,213
**Primary source of cooking**				
Smokeless	66.35	14,532	65.84	13,733
Smoked	33.65	7,370	34.16	7,124
**Owned house**				
No	5.86	1,283	13.04	2,720
Yes	94.14	20,619	86.96	18,137
**Place of residence**				
Urban	44.72	9,794	44.92	9,368
Rural	55.28	12,108	55.08	11,489
**Regions**				
Northern	20.34	4,454	20.81	4,340
North-Eastern	9.90	2,169	8.73	1,820
Central	14.87	3,256	14.78	3,082
Eastern	16.77	3,672	15.89	3,314
Western	14.04	3,076	14.91	3,110
Southern	24.08	5,275	24.89	5,191
**Total**	**100**	**21,902**	**100**	**20,857**

**Source:** Authors’ own calculation using 75th round of National Sample Survey data. **Abbreviations:** SC-Schedule Caste; ST-Schedule Tribe; OBC-Other Backward Caste.

### Gender gaps in poor current SRH

**Table 2** presents absolute gender gaps (%) in poor self-reported health about current health status among older adults. The overall absolute gender gap in poor SRH_current_ is 3.27%. About 4% absolute gender gaps (AGG) are observed in poor SRH_current_ among both young-old and middle-old age groups, which are higher than the oldest-old age. However, the higher educational attainment shows greater AGG in poor SRH_current_ which is 6.2%. Those who are physically-mobile have higher AGG in poor SRH_current_ than immobile. Despite that, uncovered insurance support (3.63%) has greater AGG in poor SRH_current_ than covered insurance (1.73%). Richest household income group (6.29%) has showed greater AGG in poor SRH_current_ than other household income groups. Higher AGG in poor SRH_current_ is observed among Christians (5.32%) and General caste (4.55%) than other religion or caste groups. However, those elderly who owned house has showed higher AGG in poor SRH_current_ than who do not owned. Lower AGG in poor SRH_current_ is observed in rural residence than urban. Besides that, greater AGG in good SRH_current_ is reflected among Northern region with 5.1% followed by Eastern (3.9%) and Southern (3.3%) while lowest is seen among North-eastern region (0.3%).

**Table 2:** Absolute gender gaps (%) in Self-Rated Health (SRH) about current health status among older adults in India by gender with suitable background characteristics, 2017–18 (n = 42,759).

	Self-Rated Health about current health status (SRH_Current_)				Absolute gap in SRH_Current_
Background characteristics	Men		Women		
	Excellent	Poor	Excellent	Poor	
**Age-group (in years)**					
Young-old (60–69)	12.87	87.22	8.73	91.27	4.05
Middle-old (70–79)	6.35	93.65	4.49	95.51	1.86
Oldest-old (80+)	3.42	96.58	3.01	96.99	0.41
**Marital Status**					
Currently married	10.89	89.11	8.59	91.41	2.30
Never married	0.26	99.74	1.05	98.95	-0.79
Separated or Divorced	8.41	91.59	5.92	94.08	2.49
**Economic dependency**					
Independent	14.34	85.66	13.97	86.03	0.37
Dependent	6.35	93.65	6.39	93.61	-0.04
**Educational attainment**					
No education	8.07	91.93	6.18	93.82	1.89
Primary	9.32	90.68	7.85	92.15	1.47
Secondary	14.35	85.65	12.87	87.13	1.48
Higher	17.40	82.60	11.20	88.80	6.20
**Working status**					
Yes	12.94	87.06	8.01	91.99	4.93
No	7.17	92.83	5.25	94.75	1.92
**Living arrangement**					
With Spouse	19.39	80.61	18.55	81.45	0.84
Without Spouse	15.76	84.24	17.78	82.22	-2.02
**Physical mobility status**					
Mobile	10.8	89.20	7.47	92.53	3.33
Immobile	4.69	95.31	3.83	96.17	0.86
**Communicable disease**					
No	10.47	89.53	7.21	92.79	3.26
Yes	6.90	93.10	3.14	96.86	3.76
**Chronic diseases**					
No	12.17	87.83	8.43	91.57	3.74
Yes	4.26	95.74	2.77	97.23	1.49
**Any other ailments**					
No	10.49	89.51	7.26	92.74	3.23
Yes	9.13	90.87	5.33	94.67	3.80
**Hospitalization**					
No	10.86	89.14	7.46	92.54	3.40
Yes	4.65	95.35	2.43	97.57	2.22
**Insurance coverage**					
Covered	7.45	92.55	5.72	94.28	1.73
Uncovered	11.11	88.89	7.48	92.52	3.63
**Household Income**					
Poorest	9.07	90.93	5.90	94.10	3.17
Poorer	9.74	90.26	6.38	93.62	3.36
Middle	9.75	90.25	9.28	90.72	0.47
Richer	9.89	90.11	7.00	93.00	2.89
Richest	13.65	86.35	7.36	92.64	6.29
**Religion**					
Hindus	10.51	89.49	7.12	92.88	3.39
Muslims	9.39	90.61	7.40	92.60	1.99
Christians	12.4	87.60	7.08	92.92	5.32
Others	9.54	90.46	7.11	92.89	2.43
**Caste groups**					
General	12.01	87.99	7.46	92.54	4.55
SC	9.20	90.80	6.86	93.14	2.34
ST	8.29	91.71	5.86	94.14	2.43
OBC	10.15	89.85	7.46	92.54	2.69
**Household Size**					
< = 5	9.94	90.06	6.92	93.08	3.02
>5	11.05	88.95	7.50	92.50	3.55
**Primary source of cooking**					
Smokeless	11.76	88.24	8.42	91.58	3.34
Smoked	8.50	91.50	5.31	94.69	3.19
**Owned house**					
No	5.57	94.43	4.51	95.49	1.06
Yes	10.71	89.29	7.54	92.46	3.17
**Place of residence**					
Urban	12.72	87.28	8.67	91.33	4.05
Rural	9.30	90.70	6.39	93.61	2.91
**Regions**					
Northern	11.22	88.70	6.11	93.80	5.10
North-Eastern	9.70	90.30	9.39	90.60	0.30
Central	8.78	91.20	6.14	93.80	2.60
Eastern	7.48	92.50	3.55	96.40	3.90
Western	15.55	84.40	12.54	87.40	3.00
Southern	10.48	89.50	7.16	92.80	3.30
**Total**	**10.42**	**89.58**	**7.15**	**92.85**	**3.27**

**Source:** Authors’ own calculation using 75th round of National Sample Survey data. **Abbreviations:** SC-Schedule Caste; ST-Schedule Tribe; OBC-Other Backward Caste. **Notes:** Chi-square tests were significant at P < .0001.

### Gender gaps in worse change in SRH

**Table 3** presents absolute gender gaps (%) in change in SRH among older adults in India from 2017–18. The overall absolute gender gap (AGG) in worse change in self-rated health status (SRH_change_) was 0.58%. Around 1.3% AGG in worse SRH_change_ are found among middle-old which is greater than the young-old (0.29%). Older adults who are currently married 1.07% has higher AGG in worse SRH_change_. Interestingly, older adults with higher educational attainment shows greatest AGG in worse SRH_change_ with 11.31%. Older adults who can physically mobile (0.98%), suffered from communicable diseases (9.62%) and other ailments (5.84%) showed higher AGG in worse SRH_change_. Older adults who do not have health insurance support and belonging to richer household income group have higher AGG in worse SRH_change_. Greater AGG in worse SRH_change_ are seen among older adults belonging to Muslim religion (2.94%) and general caste (2.94%) respectively. Older adults with household size more than five members have higher AGG in worse SRH_change._Those older adults who do not owned house have greater AGG in worse SRH_change_ than who owned house. Older adults who use smoke-as a primary source of energy for cooking in the household has greater AGG in worse SRH_change._ Again, Northern region showed higher AGG in worse SRH_change_ than other regions respectively.

**Table 3:** Absolute gender gaps (%) in Self-Rated Health (SRH) about change in health status among older adults in India by gender with suitable background characteristics, 2017–18 (n = 42,759).

Background characteristics	Self-Rated Health about change in health status (SRH_Change_)				Gap in SRH_Change_
	Men		Women		
	Better	Worse	Better	Worse	
**Age-group (in years)**					
Young-old (60–69)	19.90	80.10	19.61	80.39	0.29
Middle-old (70–79)	17.30	82.70	16.00	84.00	1.30
Oldest-old (80+)	13.19	86.81	13.37	86.63	-0.18
**Marital Status**					
Currently married	19.60	80.40	18.53	81.47	1.07
Never married	8.32	91.68	13.52	86.48	-5.20
Separated or Divorced	14.74	85.26	17.85	82.15	-3.11
**Economic dependency**					
Independent	20.45	79.55	24.74	75.26	-4.29
Dependent	16.95	83.05	17.42	82.58	-0.47
**Educational attainment**					
No education	16.89	83.11	16.75	83.25	0.14
Primary	17.70	82.30	21.86	78.14	-4.16
Secondary	23.41	76.59	24.16	75.84	-0.75
Higher	22.12	77.88	10.81	89.19	11.31
**Working status**					
Yes	20.56	79.44	19.01	80.99	1.55
No	16.38	83.62	16.26	83.74	0.12
**Living arrangement**					
With Spouse	10.93	89.07	8.66	91.34	2.27
Without Spouse	8.08	91.92	5.78	94.22	2.30
**Physical mobility status**					
Mobile	18.93	81.07	17.95	82.05	0.98
Immobile	15.81	84.19	20.18	79.82	-4.37
**Communicable disease**					
No	18.64	81.36	18.2	81.80	0.44
Yes	24.58	75.42	14.96	85.04	9.62
**Chronic diseases**					
No	20.16	79.84	19.65	80.35	0.51
Yes	13.73	86.27	13.01	86.99	0.72
**Any other ailments**					
No	18.56	81.44	18.29	81.71	0.27
Yes	21.59	78.41	15.75	84.25	5.84
**Hospitalization**					
No	18.71	81.29	18.10	81.90	0.61
Yes	19.03	80.97	18.80	81.20	0.23
**Insurance coverage**					
Covered	16.57	83.43	17.35	82.65	-0.78
Uncovered	19.24	80.76	18.33	81.67	0.91
**Household Income**					
Poorest	17.69	82.31	15.99	84.01	1.70
Poorer	16.88	83.12	16.73	83.27	0.15
Middle	18.66	81.34	20.86	79.14	-2.20
Richer	20.24	79.76	17.74	82.26	2.50
Richest	20.21	79.79	19.70	80.30	0.51
**Religion**					
Hindus	18.86	81.14	18.56	81.44	0.30
Muslims	18.37	81.63	15.43	84.57	2.94
Christians	18.08	81.92	18.03	81.97	0.05
Others	17.27	82.73	16.33	83.67	0.94
**Caste groups**					
General	19.28	80.72	16.34	83.66	2.94
SC	17.53	82.47	16.37	83.63	1.16
ST	15.99	84.01	15.47	84.53	0.52
OBC	19.62	80.38	20.85	79.15	-1.23
**Household Size**					
< = 5	19.03	80.97	19.18	80.82	-0.15
>5	18.34	81.66	16.61	83.39	1.73
**Primary source of cooking**					
Smokeless	20.88	79.12	20.47	79.53	0.41
Smoked	15.66	84.34	14.80	85.20	0.86
**Owned house**					
No	13.71	86.29	11.67	88.33	2.04
Yes	19.04	80.96	19.10	80.90	-0.06
**Place of residence**					
Urban	21.04	78.96	20.12	79.88	0.92
Rural	17.62	82.38	17.17	82.83	0.45
**Regions**					
Northern	16.24	83.76	13.09	86.91	3.15
North-Eastern	18.51	81.49	19.54	80.46	-1.03
Central	17.12	82.88	15.09	84.91	2.03
Eastern	11.35	88.65	13.68	86.32	-2.33
Western	23.91	76.09	21.88	78.12	2.03
Southern	24.35	75.65	23.45	76.55	0.90
**Total**	**18.73**	**81.27**	**18.15**	**81.85**	**0.58**

**Source:** Authors’ own calculation using 75th round of National Sample Survey data. **Abbreviations:** SC-Schedule Caste; ST-Schedule Tribe; OBC-Other Backward Caste. **Notes:** Chi-square tests were significant at P < .0001.

### Determinants of poor SRH_current_ and worse SRH_change_

**Table 4** presents the result of binary logistic regression analysis of poor SRH_current_ (Model 1) & worse SRH_change_ (Model 2) among older adults in India with suitable background characteristics, 2017–18.

**Table 4:** Binary logistic regression results for current and change in self-rated health among older adults in India by gender with suitable background characteristics, 2017–18. (n = 42,759).

Background characteristics	(Model 1)			(Model 2)		
	Current SRH			Change in SRH		
	Adjusted Odds ratio	Conf. Intervals		Adjusted Odds ratio	Conf. Intervals	
		Lower	Upper		Lower	Upper
**Gender**						
Men®						
Women	1.09*	0.99	1.19	1.09***	1.02	1.16
**Age-group (in years)**						
Young-old (60–69)®						
Middle-old (70–79)	1.81***	1.64	2.00	1.23***	1.16	1.31
Oldest-old (80+)	2.43***	1.96	3.00	1.44***	1.29	1.60
**Marital Status**						
Currently married®						
Never married	2.09**	1.04	4.19	1.07	0.75	1.53
Separated or Divorced	0.96	0.80	1.17	0.97	0.86	1.10
**Economic dependency**						
Independent®						
Dependent	1.98***	1.81	2.16	1.08**	1.01	1.15
**Educational attainment**						
No education®						
Primary	0.85***	0.77	0.93	0.95*	0.89	1.01
Secondary	0.69***	0.61	0.78	0.88***	0.81	0.95
Higher	0.55***	0.47	0.64	0.82***	0.73	0.91
**Working status**						
Yes®						
No	1.44***	1.33	1.57	1.13***	1.07	1.20
**Living arrangement**						
With Spouse®						
Without Spouse	1.09	0.91	1.32	1.06	0.94	1.19
**Physical mobility status**						
Mobile®						
Immobile	1.77***	1.43	2.18	1.26***	1.14	1.39
**Communicable disease**						
No®						
Yes	0.74**	0.57	0.96	1.11	0.93	1.32
**Chronic diseases**						
No®						
Yes	3.36***	2.96	3.81	1.76***	1.65	1.88
**Any other ailments**						
No®						
Yes	1.43***	1.17	1.74	1.11*	0.98	1.26
**Hospitalization**						
No®						
Yes	2.25***	2.02	2.51	0.84***	0.79	0.89
**Insurance coverage**						
Covered®						
Uncovered	0.87***	0.79	0.95	0.86***	0.80	0.92
**Household Income**						
Poorest®						
Poorer	0.99	0.87	1.13	0.99	0.90	1.08
Middle	0.95	0.83	1.08	0.99	0.91	1.09
Richer	0.94	0.82	1.07	0.93	0.85	1.02
Richest	0.78***	0.68	0.91	0.92	0.83	1.02
**Religion**						
Hindus®						
Muslims	1.20***	1.05	1.36	1.16***	1.06	1.26
Christians	0.94	0.80	1.11	0.97	0.86	1.09
Others	1.01	0.85	1.21	1.04	0.92	1.18
**Caste groups**						
General®						
SC	0.85**	0.73	0.99	0.90**	0.81	1.00
ST	1.03	0.91	1.16	1.02	0.94	1.11
OBC	0.92*	0.84	1.01	0.94*	0.89	1.00
**Household Size**						
< = 5®						
>5	0.81***	0.75	0.88	1.00	0.95	1.06
**Primary source of cooking**						
Smokeless®						
Smoked	1.22***	1.11	1.34	1.26***	1.18	1.34
**Owned house**						
No®						
Yes	0.88*	0.75	1.02	0.84***	0.76	0.92
**Place of residence**						
Urban®						
Rural	1.03	0.94	1.13	1.09***	1.02	1.16
**Regions**						
Northern®						
North-Eastern	0.98	0.84	1.14	0.88**	0.79	0.98
Central	1.08	0.94	1.23	0.87***	0.79	0.95
Eastern	1.46***	1.27	1.69	1.21***	1.09	1.33
Western	0.58***	0.52	0.65	0.62***	0.57	0.67
Southern	0.73***	0.65	0.83	0.57***	0.52	0.62

***Source*:** *Authors’ own calculation using 75th round of National Sample Survey data*. **Abbreviations:** SC-Schedule Caste; ST-Schedule Tribe; OBC-Other Backward Caste; AOR-Adjusted odds ratio; C.I.- confidence interval. **Notes:** Self-Rated Health (SRH) about current health status is the dependent variable for model 1; Self-Rated Health (SRH) about change in health status is another dependent variable indicated by Model 2; confidence interval in the parentheses; Significant level at: *** significant at 1 percent, ** significant at 5 percent and * significant at 10 percent; ® is the reference category of the independent variables.

**Model 1** in Table **4** presents that poor SRH_current_ versus excellent are found to be significantly greater among older women [AOR = 1.09; CI = 0.99, 1.19] than older men. The middle-old [AOR = 1.81; CI = 1.64, 2.00] and oldest-old [AOR = 2.43; CI = 1.96, 3.00] have significantly higher odds of poor SRH_current_ compared to young old. However, economically dependent older adults [AOR = 1.98; CI = 1.81, 2.16] are significantly more likely to have poor SRH_current_ compared to economically independent older adults. Older adults with primary [AOR = 0.85; CI = 0.77, 0.93], secondary [AOR = 0.69; CI = 0.61, 0.78] and higher [AOR = 0.55; CI = 0.47, 0.64] education level have significantly lower odds of poor SRH_current_ compared to no education. Physically immobile older adults [OR = 1.77; CI = 1.43, 2.18] are significantly more likely to have poor SRH_current_ compared to who can physically mobile. Lower odds of poor SRH_current_ are observed among older adults suffered with communicable diseases [AOR = 0.74; CI = 0.57, 0.96] while greater odds of poor SRH_current_ are seen with chronic diseases [AOR = 3.36; CI = 2.96, 3.81]. However, significantly greater odds of poor SRH_current_ are seen among older adults who have been hospitalized [AOR = 2.25; CI = 2.02, 2.51]. On the other hand, older adults who are not covered with any health insurance [AOR = 0.87; CI = 0.79, 0.95] and belonging to richest income group [OR = 0.78; CI = 0.68, 0.91] have lower odds of poor SRH_current_. Muslims [AOR = 1.20; CI = 1.05, 1.36] are significantly more likely to have poor SRH_current_ compared to Hindus. While Schedule caste [AOR = 0.85; CI = 0.73, 0.99] and OBC [AOR = 0.92; CI = 0.84, 1.01] are less likely to have poor SRH_current_ compared to General caste. However, Eastern region [AOR = 1.46; CI = 1.27, 1.69] are significantly more likely to have poor SRH_current_ while Western [AOR = 0.58; CI = 0.52, 0.65] and Southern [AOR = 0.73; CI = 0.65, 0.83] regions are significantly less likely to have poor SRH_current_ compared to Northern region respectively.

Meanwhile, in **Table 4, Model 2** presents the result of binary logistic regression for SRH_change_ among older adults in India. We found similar finding as seen in the model 1, where older women, middle-old, oldest-old, economically dependent, physically immobile, working older adults are significantly more likely to have worse SRH_change_. While older adults with primary, secondary and higher educational level, Schedule caste and OBC have lower odd of worse SRH_change_. Older adults who suffered from chronic diseases and other ailments were more likely to have worse SRH_change_. Lower odds of worse SRH_change_ have been observed among older adults who were hospitalized and those who were not covered by health insurance. Muslim religion [AOR = 1.16; CI = 1.06, 1.26] has also found to have higher odds of worse SRH_change_ compared to Hindus. Compared to participants in urban residence, those in rural residence [AOR = 1.09; CI = 1.02, 1.16] had higher odds of worse SRH_change_. However, Southern, Western, Central and North-eastern regions showed lower odds of worse SRH_change_ while the Eastern region [AOR = 1.21; CI = 1.09, 1.33] show higher odds of worse SRH_change_ than the Northern region.

## Discussion

We have used India’s large-scale national sample survey data, where we have examined not only the current SRH but also analyzed it to study the change in SRH among older adults from a gender perspective. In support of the sponge hypothesis, our finding revealed that there are substantial gender gaps among older Indian adults with a female disadvantage in both poor SRH_current_ and worse SRH_change_. Older women are significantly more likely to have poor SRH_current_ and worse SRH_change_ compared to older men and our finding is consistent with the previous studies [11, 13, 17, 38].

Our findings indicate that several demographic factors such as different age-groups of older adults, marital status, educational level, religion, caste, place of residence, geographical regions have played a substantial role in impacting both poor SRH_current_ and worse SRH_change_. We found that middle-old (70–79 years) and oldest-old (80+ years) are more likely to have both poor SRH_current_ and worse SRH_change_, compared to young-old (60–69 years). While a previous study [17] has documented that only oldest-old (80+) were having greater poor SRH compared to young-old. Our findings suggest that older adults who are never married are significantly have greater poor SRH_current_ compared to currently married older adults and similar study has been depicted in recent study conducted in China [39].

The results from our analysis confirmed the findings from the previous research that older adults who were economically dependent had a higher risk of having poor SRH [17, 18, 40]. Our findings found that older adults who are physically immobile have poor SRH_current_ and worse SRH_change_ compared to older adults who are physically mobile and similar results are also observed in previous studies [18, 19]. Meanwhile, our findings also revealed that older adults who are covered with health insurance support have higher chances of poor SRH_current_ and worse SRH_change_ compared to older adults who are uninsured and earlier study conducted in Jamaica has also depicted similar findings [41]. Previous study [18] has found that there exists positive association between living arrangements and SRH but our finding showed no statistically significant association between living arrangement and SRH.

Morbidity is a strong predictor of poor SRH among older adults in India [18]. Our finding revealed that older adults suffering from chronic diseases have a greater risk of poor SRH_current_ and worse SRH_change_, compared to older adults who are not suffering from any chronic diseases, while earlier study has also confirmed the similar findings [18]. Poor SRH_current_ and worse SRH_change_ are strongly associated with hospitalizations, our findings conformed from the recent study [24] that older adults who are hospitalized have higher risk of poor SRH_current_ and lower risk of worse SRH_change_.

Literature suggests that there is an inverse relationship between educational level and poor SRH and our study showed similar findings [11, 17, 42]. Previous studies [17, 42] have emphasized that religion and social groups-for instance Muslims and SCs have greater risk of poor SRH than other reference groups. Similarly, multiple previous studies documented the examples of diminished returns theory [43–45], where factors such as race can reduce the return of socioeconomic advantages on individuals’ SRH. However, our study only showed similar finding in term of religious groups. On the other hand, our findings found that older adults belonging to the SC group had significantly lower odds of poor SRH_current_ compared to General caste group which contradicts with the previous studies [17, 42]. Our findings also revealed that older adults belonging to rural residence have greater odds of worse SRH_change_, as a result, in rural residence, there is a dearth of sufficient health care facilities and other critical civic services, as well as sociocultural and changing family customs. Our findings suggest that there is a need to improve health-related infrastructure in rural regions which can be an effective approach to bringing an equitable health and wellbeing among older populations in the country.

Furthermore, our findings clearly suggest that older people belonging to Eastern region are significantly more likely to have poor SRH_current_ and worse SRH_change_ compared to their peers in Northern region. Meanwhile, variations in poor SRH among older adults across the country may be related to the diversity of areas in terms of resource availability and the condition of socioeconomic and demographic advancement. Previous studies showed that when compared to other regions, the states included in the Central and Eastern regions have below-average socioeconomic and demographic factors [17, 18]. The primary health care infrastructure in these states is below average and accessibility to these facilities is also not universal [17].

Additionally, Ministry of Social Justice & Empowerment of India has recommended the National Council for Older Persons (NCOP) to strengthened the various amendments and programs provided by them [46]. While NCOP has intervened in several aging-related concerns, including pensions, travel concessions, income tax reliefs, medical and health care benefits, and other perks that would eventually help people maintain a higher level of life. The council has asked social scientists and health professionals to identify important challenges affecting India’s older population. However, this study could provide an insight for future health policies and initiatives.’

## Limitations

Our study has several limitations. First, our study is based on a cross-sectional survey, which eliminates the possibility of temporal ambiguity for drawing causal inferences. Second, we did not include the other key factors while examining the self-rated health status- like body mass index, frailty, and other nutritional health outcomes could not be examined since the data was not available about them in the sample taken for consideration. Third, other personal habits factors such as smoking, drinking alcohol, chewing tobacco are not included because of the data unavailability. Lastly, we have also not included the lifestyle factors which also an important predictor of SRH.

## Conclusions

Out study has addressed the significant public health concern, which is key to addressing the challenge of older adults’ health and their perception of well-being. Supporting the sponge hypothesis, a clear gender gap was observed in poor current SRH and worse change in SRH among older adults in India with a female disadvantage. We further found lower socioeconomic and health conditions and lack of resources as determinants of poor current SRH and its worse change among older Indians. Older adults are more vulnerable to health and physical outcomes given the age-related life cycle changes, so the increased risk for active and healthy aging is likely a challenge given the low perception about current health status. Moreover, the challenges are multiple given the asymmetry from a gender perspective since women are more prone to these health outcomes, which likely risks their well-being. Therefore, this study identifies a significant gender gap in this domain since identifying older adults’ health perception can be significant in terms of their healthcare services and caregiving approaches.

## Abbreviations

SRH: Self-rated health
SRH_current_: Current self-rated health status
SRH_change_: Change in self-rated health status
NSSO: National Sample Survey Organizations
MOSPI: Ministry of Statistics and Programme Implementation
UT: Union Territories
AGG: Absolute gender gap
SC: Schedule Caste
ST: Schedule Tribe
OBC: Other Backward Caste
AOR: Adjusted odds ratio
C.I: confidence interval
