# Supplementary material for: Self-rated health among older adults in India: Gender specific findings from National Sample Survey
Source: PLoS One. 2023 Apr 17;18(4):e0284321. doi: 10.1371/journal.pone.0284321 (PMC10109469; doi:10.1371/journal.pone.0284321)
Supplement: Attachment — Submitted filename: Response to reviewers.docx [file pone.0284321.s001.docx]

**Response to reviewers**

**Reviewer 1:**

**Comment 1:** There is a literature on sponge hypothesis, that SRH may differently reflect health of men and women. Here are some example publications:

<https://pubmed.ncbi.nlm.nih.gov/32395609/>

**Authors ’Response 1:** Thank you for the comment. The sponge hypothesis has been introduced now in the manuscript and the explanations are provided for the hypothesis by citing the suggested references.

**Comment 2:** There are also papers showing gender have different roles on SRH across countries such as India and other countries. This means, local data are necessary, because what is relevant to India is specific to India and would not inform policies in other regions. Thus, India would need local data for local valid policy making.

<https://pubmed.ncbi.nlm.nih.gov/27651902/>

**Authors ’Response 2:** We appreciate your valuable suggestions. The background section is revised accordingly and the objectives and rationale of the study are modified now, as per suggestion. The above reference along with other references has been cited now.

**Comment 3:** You found that "Respondents belonging to richest income quintile and not covered by any health insurance, belonging to Schedule caste, OBC, Western and Southern regions are found to have poor SRHcurrent and worse SRHchange." This could be discussed via marginalization-related diminished returns theory, that reduces the return of SES such as income in the presence of any marginalizing factor. Here are some example papers on diminished returns of income or education on self-rated health:

<https://pubmed.ncbi.nlm.nih.gov/?term=diminished+returns+self-rated+health+income&size=20>

**Authors ’Response 3:** Done! Explanations of some of the findings are provided based on diminished returns theory and the suggested references are cited now in the discussion section. Kindly note that there was a typo in the above statement in the abstract and it is corrected now.

**Comment 4:** I think some of the discussion should be on marginalization that reduces the return of SES such as income.

**Authors ’Response 4:** Done! Thank you for the suggestion.

**Comment 5:** English needs some additional work. Someone should edit the paper.

**Authors ’Response 5:** The manuscript is proofread multiple times by the authors and as a result, it has improved now after revising for typos, grammatical errors and the English language. We appreciate your reviewing our manuscript and thank you for your time.
